# Robotic arm‐assisted total knee arthroplasty reduces postoperative inflammatory response and blood loss compared to manual total knee arthroplasty: A matched‐pairs analysis of 688 patients

**DOI:** 10.1002/ksa.70054

**Published:** 2025-09-09

**Authors:** Dirk Müller, Igor Lazic, Benjamin Schloßmacher, Vincent Lallinger, Michael T. Hirschmann, Rüdiger von Eisenhart‐Rothe, Florian Pohlig

**Affiliations:** ^1^ Department of Orthopaedic Surgery, School of Medicine, Klinikum rechts der Isar Technical University of Munich Munich Germany; ^2^ Department of Orthopaedic Surgery and Traumatology Kantonsspital Baselland (Bruderholz, Liestal, Laufen) Bruderholz Switzerland; ^3^ Schoen Clinic Munich Munich Germany

**Keywords:** blood loss, C‐reactive protein (CRP), manual total knee arthroplasty (mTKA), matched‐pairs analysis, robotic arm‐assisted total knee arthroplasty (raTKA), systemic inflammatory response

## Abstract

**Purpose:**

Robotic arm‐assisted total knee arthroplasty (raTKA) has demonstrated several advantages over manual TKA (mTKA), including enhanced early recovery. Reduced soft tissue trauma and avoidance of femoral intramedullary canal opening have been hypothesised to lower the systemic inflammatory response. However, findings from previous small‐cohort studies have been inconsistent. This study aimed to evaluate postoperative systemic inflammation in a large patient cohort.

**Methods:**

Patients who underwent raTKA using the Mako® system were matched with patients who received mTKA based on gender, American Society of Anesthesiologists score, age and body mass index. This matching process resulted in two comparable cohorts, each comprising 344 patients. Blood samples were collected preoperatively, 6 h postoperatively, and on postoperative Days 3 and 5. Measurements included C‐reactive protein (CRP), white blood cell count (WBC) and calculated blood loss.

**Results:**

The highest CRP levels were observed on postoperative Day 3 in both groups. The median CRP increase was significantly lower in the raTKA group compared with the mTKA group on Day 3 (4.4 vs. 5.3 mg/dL; *p* = 0.002) and slightly lower on Day 5 (3.5 vs. 3.8 mg/dL; *p* = 0.349). The WBC count peaked at 6 h postoperatively in both groups before steadily declining, with no significant difference between groups. The median operation time was significantly longer in the raTKA group (92 vs. 86 min; *p* < 0.001). Despite a longer surgical duration, the median blood loss was significantly lower in the raTKA group (653 vs. 729 mL; *p* = 0.005).

**Conclusion:**

In the largest comparative analysis to date, raTKA was linked to significantly lower postoperative CRP levels than mTKA. Reduced soft tissue trauma, avoidance of femoral intramedullary canal violation and significantly lower blood loss may all contribute to a diminished systemic inflammatory response, potentially explaining the improved early functional outcomes observed with raTKA.

**Level of Evidence:**

Level III, retrospective comparative study.

**Abbreviations**
ASA: American Society of Anesthesiologists
BMI: body mass index
CR: cruciate retaining
CRP: C‐reactive protein
Hb: haemoglobin
mTKA: manual total knee arthroplasty
PS: posterior stabilised
PSI: patient specific instrumentation
raTKA: robotic arm‐assisted total knee arthroplasty
TKA: total knee arthroplasty
WBC: white blood cell count

## INTRODUCTION

Total knee arthroplasty (TKA) is a well‐established and highly effective treatment for end‐stage knee osteoarthritis. In recent years, robotic arm‐assisted TKA (raTKA) has become a major trend, offering improved accuracy in limb alignment and implant positioning compared with conventional manual TKA (mTKA) [33, 38, 44]. In addition, patient‐specific approaches such as kinematic or functional alignment strategies are increasingly used, minimising the need for extensive soft tissue release [3, 36]. Benefits such as reduced postoperative pain [20], improved early functional recovery and shorter hospital stays [14, 23, 24] have been observed with raTKA. Furthermore, better patient‐reported outcomes [1, 44] and higher forgotten joint scores have been reported for raTKA [12]. A potential explanation for these findings is a lower postoperative systemic inflammatory response, which could be attributed to reduced soft tissue trauma associated with raTKA [9, 17]. Kayani et al. demonstrated a significantly lower degree of soft tissue trauma in raTKA compared with mTKA [13]. The avoidance of femoral intramedullary canal opening in raTKA is considered a further reason for a lower systemic inflammatory response. Notably, navigation‐assisted TKA, which avoids femoral intramedullary rods, has shown significantly lower peak CRP levels than mTKA [2, 34].

While some evidence suggests that a reduced postoperative systemic inflammatory response may play a crucial role in the improved early recovery seen in raTKA compared with mTKA, previous studies involving small cohorts have reported conflicting results [15, 20, 21, 41, 42, 43]. This large‐cohort study tests the hypothesis that patients undergoing raTKA exhibit a lower postoperative systemic inflammatory response than those undergoing mTKA.

## MATERIALS AND METHODS

### Patient selection and matching procedure

**Figure 1:**
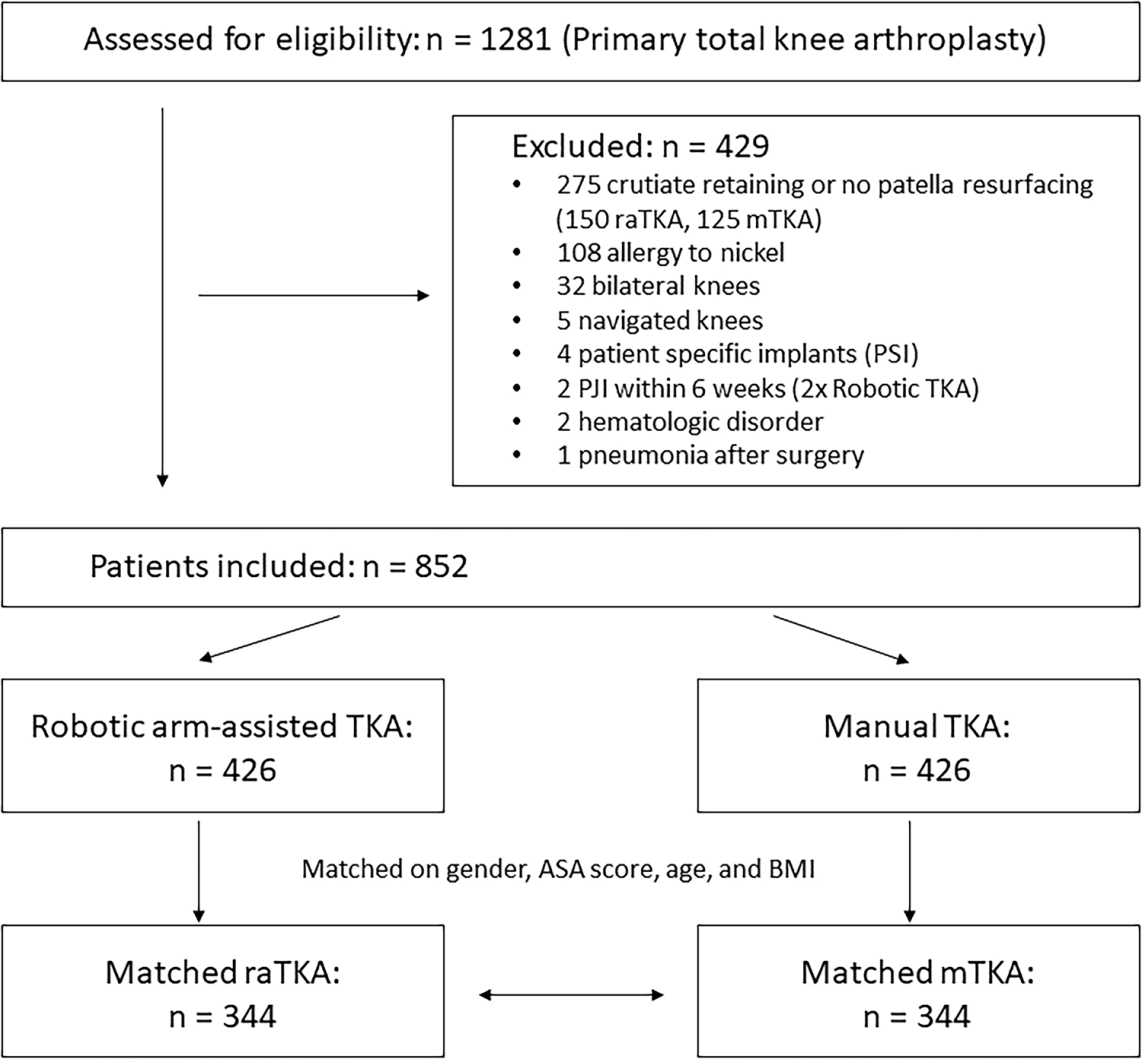
Flowchart of patient selection and matching procedure. mTKA, manual total knee arthroplasty. raTKA, robotic arm‐assisted total knee arthroplasty.

Patients treated with unilateral TKA between October 2019 and June 2024 were included in this retrospective study. Exclusion criteria included the use of revision implants, patient‐specific implants, metal hypersensitivity, postoperative surgical site infection, infection of other organs and haematologic disorders. The use of posterior‐stabilised (PS) implants and patellar resurfacing was the standard at our institution. In order to increase comparability, patients treated with cruciate‐retaining (CR) implants or without patellar resurfacing were excluded. Patients were allocated to the mTKA or raTKA group based on patient preference. To reduce confounding and ensure comparability between treatment groups, a matched‐pairs analysis was performed. Patients who underwent raTKA were matched with mTKA patients in a 1:1 ratio using exact matching on gender and ASA score. Continuous covariates were matched with a caliper of ±7 years for age and ±3 kg/m² for BMI. Matching was performed using the Hungarian algorithm and executed without replacement. This method yielded a final set of 344 matched pairs with improved balance across covariates. Matching was performed using Python 3.10. The balance between groups after matching was assessed using standardised mean differences (SMD) and Mann–Whitney *U*‐tests or chi‐squared tests. Variables used in the matching process demonstrated negligible imbalance (SMD < 0.1, *p* > 0.05), confirming the effectiveness of the matching procedure. The flowchart of patient selection and the matching procedure is shown in Figure 1, and patient characteristics are presented in Table 1.

**Table 1:** Patient characteristics.

	Robotic TKA (*n* = 344)	Manual TKA (*n* = 344)	*p*‐value	SMD
Gender (female/male, %)	54/46	54/46	1.000	0.000
Age (years at surgery, median, Q1–Q3)	69 (62–77)	69 (61–77)	0.732	0.021
BMI (kg/m², median, Q1–Q3)	27.9 (25.2–31.6)	28.4 (25.4–32.3)	0.284	−0.062
ASA (I–II/III–IV, %)	80/20	80/20	1.000	0.000
Laterality (left/right, %)	47/53	49/51	0.555	0.068
General/spinal anaesthesia (%)	93/7	93/7	1.000	0.006

*Note*: All variables demonstrated negligible imbalance (SMD < 0.1, *p* > 0.05).

Abbreviations: ASA, American Society of Anesthesiologists; BMI, body mass index; SMD, standardised mean differences; TKA, total knee arthroplasty.

### Surgical technique

raTKA was performed with the Mako® robotic system and the Triathlon® total knee system (Stryker). For mTKA, the Attune™ knee system (DePuy Synthes) was used. Preoperatively, all patients received 1 g of tranexamic acid and 1.5 g of cefuroxime intravenously. No tourniquet was used in any case. A standard midline skin incision with a medial parapatellar approach was performed in all patients.

In the manual group, a mechanical alignment strategy was applied. Tibial resection was guided by an extramedullary alignment tool, while coronal femoral alignment was achieved using an intramedullary approach. Soft tissue balance was achieved through osteophyte removal and additional soft tissue release techniques, including pie‐crusting of the medial collateral ligament and the posteromedial joint capsule.

In the raTKA group, a functional alignment strategy was implemented as described by Lustig et al. [22]. As this is based on an inverse kinematic approach, a symmetrical tibial resection was performed. Ligament tension was then assessed, and minor adjustments were made to the kinematically aligned femoral component to minimise or eliminate the need for soft tissue release. Additionally, the femoral medullary canal remained closed; however, four temporary Schanz pins were bicortically fixed in the femoral and tibial diaphysis in order to hold the navigation arrays.

All implants were fixed using vacuum‐mixed bone cement containing gentamicin (Palacos R + G, Heraeus). No surgical drains were used in any case. After joint capsule closure, 1.5 g of tranexamic acid was injected into the joint. Apixaban 2.5 mg was prescribed twice daily for 2 weeks to prevent thromboembolic events.

### Blood samples

Blood samples were collected preoperatively, 6 h postoperatively, on Day 3, and on Day 5. White blood cell count (WBC, ×10⁹/L) and haemoglobin concentration (Hb, g/dL) were measured using the Sysmex XE‐5000 analyser (Sysmex). C‐reactive protein (CRP, mg/dL) was measured preoperatively, on Day 3, and on Day 5 using the Cobas 8000 modular analyser (Roche). All three parameters (CRP, WBC and Hb) were measured and reported with an accuracy of one decimal place. For the statistical analysis of the postoperative CRP and WBC increases, the preoperative baseline values were subtracted. The intraoperative Hb loss was calculated by subtracting the postoperative haemoglobin levels (Hb_post_) from preoperative Hb levels (Hb_pre_). The intraoperative blood loss (mL) was calculated based on the intraoperative Hb loss and the blood volume. The circulating blood volume (BV) was calculated, based on the Nadler formula: [27] For males: BV (mL) = 1000 × (0.3669 × height (m)^3^ + 0.03219 × weight (kg) + 0.6041). For females: BV (mL) = 1000 × (0.3561 × height (m)^3^ + 0.03308 × weight (kg) + 0.1833). The estimated blood loss was calculated based on the haemoglobin balance method: [7, 8, 40] Estimated blood loss (mL) = Blood volume × (Hb_pre_ ‐ Hb_post_)/Hb_pre_.

### Statistical analysis

Statistical analyses were performed using SPSS software, version 29 (IBM Corporation). The normality of continuous variables was assessed with the Shapiro–Wilk test. Normally distributed variables are reported as mean ± standard deviation (SD), whereas nonnormally distributed variables are presented as median (Q1–Q3). Between‐group comparisons for continuous variables were conducted using the Student's *t*‐test or the Mann–Whitney *U*‐test, as appropriate. Categorical variables are presented as frequencies and percentages, and compared using the *χ* ^2^ test. A *p*‐value ≤ 0.05 was considered statistically significant.

Post hoc power analyses were conducted for key outcomes to evaluate the ability of the study to detect the observed group differences. For CRP increase on postoperative Day 3, the observed effect size was Cohen's *d*  = 0.21 (raTKA: *n* = 335; mTKA: *n* = 336), resulting in a calculated power of 78.8% using a two‐sided *t*‐test with *α* = 0.05. For the estimated blood loss, the observed effect size was Cohen's *d* = 0.23 (raTKA: *n* = 343; mTKA: *n* = 344), yielding a post hoc power of 85.3% under the same parameters. These findings suggest that the sample size was sufficient to detect small but potentially clinically relevant differences between groups in both outcomes.

## RESULTS

**Figure 2:**
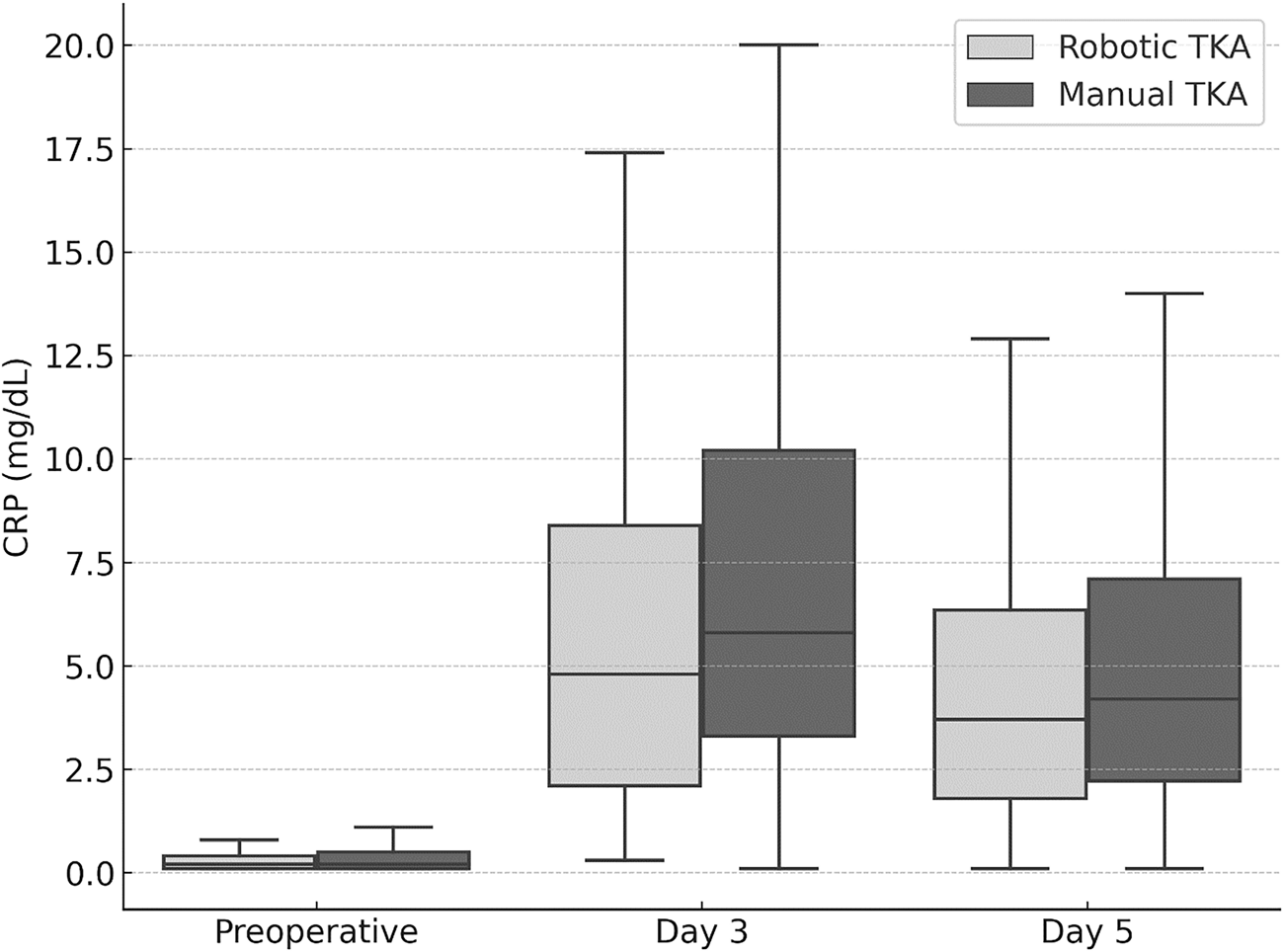
C‐reactive protein (CRP) levels (median, mg/dL) over time in robotic and manual total knee arthroplasty (TKA).

**Figure 3:**
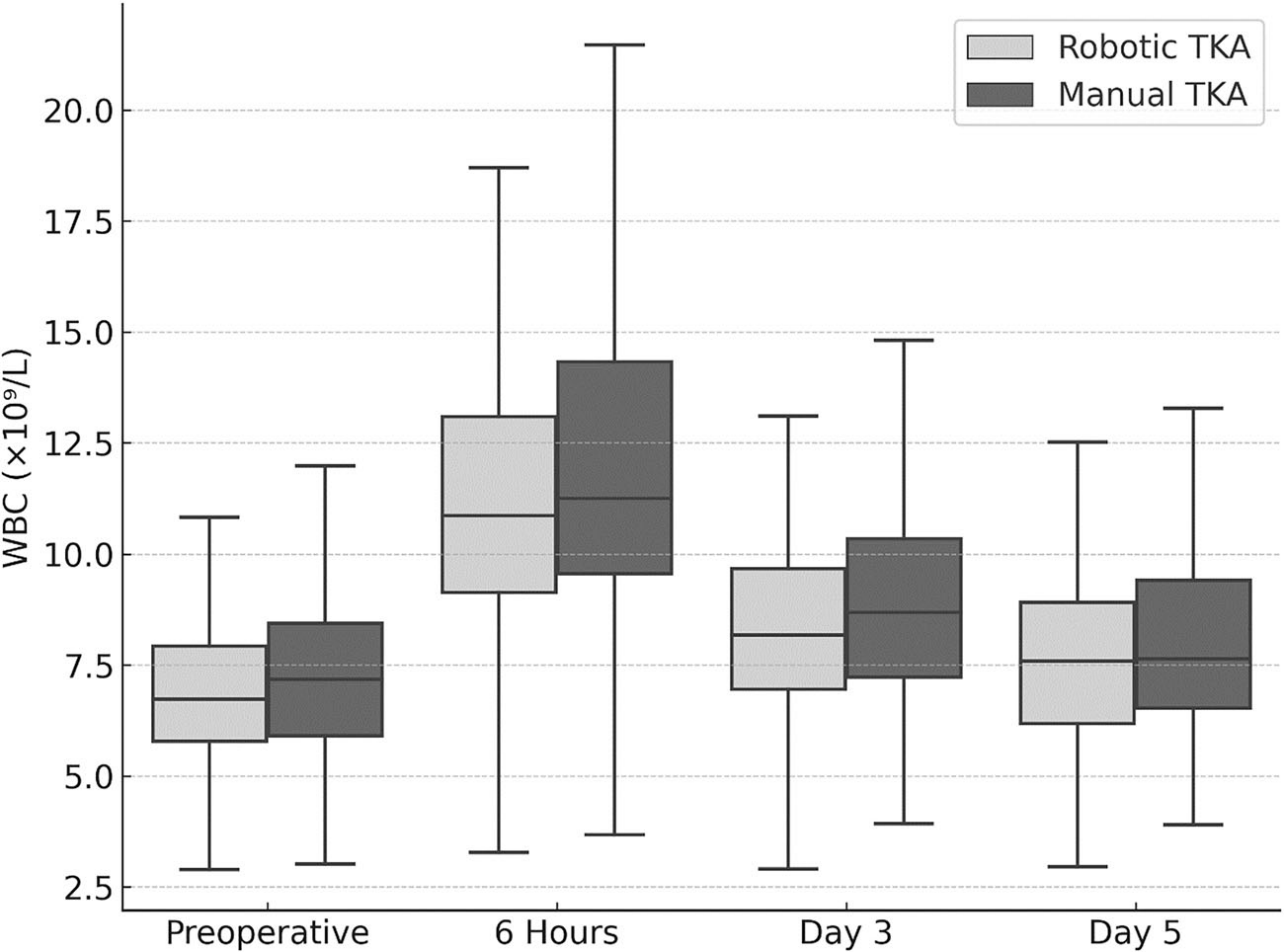
White blood cell count (WBC) levels (median, x10⁹/L) over time in robotic and manual total knee arthroplasty (TKA).

The highest CRP levels were observed on postoperative Day 3 in both groups. The peak postoperative CRP on Day 3 was significantly lower in the raTKA group compared with mTKA. The WBC count peaked at 6 h postoperatively, then declined on subsequent days in both groups (Figure 2, Table 2). No significant differences in WBC were observed between raTKA and mTKA at any time point (Figure 3, Table 2).

**Table 2:** Summary of operative time, CRP and WBC increases on Days 3 and 5, as well as haemoglobin decrease and calculated blood loss for raTKA and mTKA.

	Robotic TKA	Manual TKA	*p*‐value
Operative time (minutes), median (Q1–Q3)	92 (82−104) (*n* = 344)	86 (75−102) (*n* = 344)	<0.001
CRP increase Day 3 (mg/dL), median (Q1–Q3)	4.4 (1.9−7.9) (*n* = 335)	5.3 (3.0−9.5) (*n* = 336)	0.002
CRP increase Day 5 (mg/dL), median (Q1–Q3)	3.5 (1.4−6.1) (*n* = 111)	3.8 (1.7−6.8) (*n* = 174)	0.349
WBC increase 6 h (×10⁹/L), median (Q1–Q3)	4.2 (2.5–6.0) (*n* = 341)	4.4 (2.4−6.8) (*n* = 337)	0.297
WBC increase Day 3 (×10⁹/L), median (Q1–Q3)	1.4 (0.4−2.5) (*n* = 338)	1.6 (0.4−2.8) (*n* = 341)	0.139
WBC increase Day 5 (×10⁹/L), median (Q1–Q3)	0.5 (−0.2 to 1.8) (*n* = 112)	0.5 (−0.6 to 1.7) (*n* = 173)	0.517
Haemoglobin decrease (g/dL), median (Q1–Q3)	1.9 (1.4−2.4) (*n* = 343)	2.1 (1.5−2.6) (*n* = 344)	0.013
Blood loss (mL), median (Q1–Q3)	653 (477–882) (*n* = 343)	729 (528–938) (*n* = 344)	0.005

Abbreviations: CRP, C‐reactive protein; mTKA, manual total knee arthroplasty; raTKA, robotic arm‐assisted total knee arthroplasty; WBC, white blood cell count.

**Figure 4:**
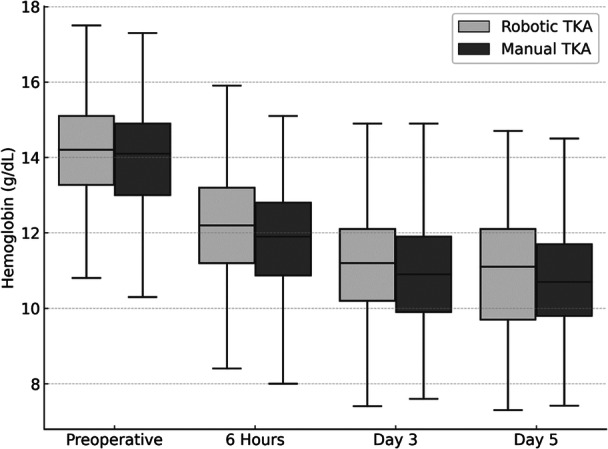
Haemoglobin levels (median, g/dL) over time in robotic and manual total knee arthroplasty (TKA).

The median operative time was significantly longer in the raTKA group (Table 2). Despite the longer operation, the decrease in haemoglobin and calculated blood loss was significantly lower in the raTKA group compared with the mTKA group (Table 2). Blood transfusions were required in 11 patients in the mTKA group (3.2%) compared with four patients in the raTKA group (1.2%), with no statistically significant difference between the groups (*p* = 0.117). The indication for transfusion was a haemoglobin level below 8.0 g/dL accompanied by clinical symptoms of anaemia. All required transfusions were administered no earlier than 6 h postoperatively, and therefore did not affect the blood loss calculation. The lowest postoperative haemoglobin levels were observed on postoperative Day 5 in both groups (Figure 4).

## DISCUSSION

The key finding of this study is that CRP levels were significantly lower following raTKA, with levels peaking on postoperative Day 3 in both groups. These results support the hypothesis that raTKA is associated with a reduced systemic inflammatory response compared with mTKA.

In line with our findings, multiple studies have reported that CRP levels typically peak on the second or third postoperative day following arthroplasty surgery [5, 11, 25, 29, 34] and return to baseline within 2 weeks [11] to 1 month [5, 25, 30]. CRP is routinely used in clinical practice to monitor postoperative recovery following TKA [26, 32]. Previous studies comparing CRP dynamics after raTKA versus mTKA largely support the results of the present study [15, 20, 21, 41, 42, 43] (Table 3). Notably, only the study by Li et al. included a cohort larger than 100 subjects; however, CRP was assessed at a single, unspecified postoperative time point, which may limit the interpretability and reliability of the findings [21]. In our cohort of 688 patients, the calculated power for CRP on postoperative Day 3 was 78.8%, indicating that prior studies were likely underpowered.

**Table 3:** Summary of previous comparative studies on postoperative systemic inflammation after raTKA and mTKA.

Author	Year	*n* Patients (raTKA/mTKA)	Robotic system	CRP	WBC
Kayani et al. [15]	2021	15/15	Mako® (Stryker)	Robotic lower CRP on Day 7, no difference on Days 2, 4, 28	‐
Li et al. [20]	2022	26/26	Not specified	No difference, day not stated	No difference, day not stated
Xu et al. [41]	2022	34/31	Mako® (Stryker)	Robotic lower CRP on Day 3, no difference on Day 1	No difference
Xu et al. [42]	2022	17/16	KUNWU® (Yuanhua)	Robotic lower CRP at 24 h, Day 3, 1 month, 2 months	Robotic lower WBC at 24 h
Li et al. [21]	2024	133/197	Mako® (Stryker)	Robotic higher CRP, day not stated	No difference, day not stated
Yuan et al. [43]	2024	28/32	KUNWU® (Yuanhua)	Robotic lower CRP on Day 3	No difference

Abbreviations: CRP, C‐reactive protein; mTKA, manual total knee arthroplasty; raTKA, robotic arm‐assisted total knee arthroplasty; WBC, white blood cell count.

In contrast to CRP levels, we did not observe lower WBC counts—a further marker of postoperative inflammation [4, 35]—following raTKA compared with mTKA. This finding aligns with the majority of previous studies [20, 21, 41, 43], with the exception of Xu et al., who reported reduced WBC levels in the raTKA group [42].

Reduced soft tissue trauma and the avoidance of bone marrow injury associated with intramedullary femoral alignment in mTKA are considered key factors contributing to the lower CRP levels observed in patients undergoing raTKA. In this context, Kayani et al. [13] proposed a classification system for iatrogenic bone and periarticular soft tissue injury in knee arthroplasty, demonstrating that raTKA is associated with significantly less soft tissue damage. This has been attributed to more personalised implant alignment, reduced need for soft tissue releases [3, 36], and improved protection of surrounding structures [9, 17]. Among these factors, avoidance of femoral medullary canal violation appears to be particularly critical [19]. Neumaier et al. [28] showed that CRP levels were significantly higher after hip arthroplasty than after dynamic hip screw procedures, while no difference was found when compared with proximal femoral nailing—suggesting that bone marrow involvement plays a central role. Supporting this hypothesis, Fontalis et al. [6] demonstrated significantly lower interleukin levels in drain fluid following raTKA compared with mTKA.

Similar to raTKA, navigated TKA does not require opening the femoral medullary canal, suggesting it may also be associated with a reduced systemic inflammatory response compared with mTKA. Previous studies have confirmed lower postoperative CRP peak levels following navigated TKA [2, 34], although the sample sizes were relatively small. Additionally, Kuo et al. demonstrated significantly lower serum levels of inflammatory cytokines and reduced interleukin levels in drainage fluid in navigated TKA [18]. Patient‐specific instrumentation (PSI) in TKA also avoids violating the femoral canal. However, Thienpont et al. found no significant differences in postoperative CRP levels between PSI and mTKA [39].

Surgical duration appears to play only a minor role in the development of the systemic inflammatory response following TKA. In the present study, operative times were significantly longer for raTKA compared with mTKA. These findings are consistent with the majority of previously published studies, which also reported longer operative times for raTKA [20, 21, 41, 42, 43].

Interestingly, despite the longer surgical duration in the raTKA group, we observed significantly lower blood loss compared with mTKA. This may be attributable to the avoidance of femoral intramedullary canal violation in raTKA. Consistent with our findings, Onggo et al. also reported significantly lower blood loss in raTKA, despite a longer operative time, in their meta‐analysis [31]. Supporting this observation, Kayani et al. [14] and Khan et al. [16] reported significantly lower blood loss following raTKA. Maman et al. further supported these findings by reporting lower transfusion rates in raTKA [23, 24]. In contrast, other studies [21, 43] found no notable difference in intraoperative blood loss between techniques, though these were limited by small sample sizes. In a retrospective study involving 486 patients, Stimson et al. reported no significant difference in Hb decrease between raTKA using the Mako® system and mTKA [37]. However, the study's interpretation is limited by the use of Hb decrease alone, without calculation of actual blood loss. Our data further demonstrate that postoperative Hb levels typically reach their nadir on postoperative Day 5, a finding of particular relevance for fast‐track and outpatient TKA protocols, where patients are frequently discharged before this point.

## LIMITATIONS

This study has several limitations. First, patient allocation to the robotic and manual TKA groups was based on patient preference rather than randomisation, which introduces the possibility of selection bias. Second, different implant systems were used in the two groups, potentially influencing outcomes and limiting the comparability of results. Third, this study utilised an image‐based raTKA system; therefore, the findings may not be directly applicable to imageless robotic systems, which could yield different results [10]. Fourth, although the differences in CRP levels and blood loss were statistically significant, they were relatively small; thus, the clinical relevance of these findings remains uncertain.

## CONCLUSION

In conclusion, this study represents the largest to date comparing systemic inflammatory responses between robotic‐assisted and manual TKA. Our matched cohort analysis demonstrated that raTKA is associated with significantly lower postoperative CRP levels and reduced blood loss, despite longer operative times. These findings suggest that the tissue‐sparing nature of raTKA may help mitigate surgical trauma and its physiological effects. Overall, the results support the potential of robotic assistance to not only improve surgical precision but also reduce the biological impact of TKA.

## AUTHOR CONTRIBUTIONS

**Dirk Müller**: Conceptualisation; methodology; data collection; analysis, interpretation of patient data and statistics; writing—original draft preparation. **Igor Lazic**: Analysis; interpretation of patient data and statistics; writing—review and editing. **Benjamin Schloßmacher**: Writing—review and editing. **Vincent Lallinger**: Writing—review and editing. **Michael T. Hirschmann**: Writing—review and editing. **Rüdiger von Eisenhart‐Rothe**: Writing—review and editing. **Florian Pohlig**: Conceptualisation and methodology; analysis; interpretation of patient data and statistics; writing—original draft preparation. All authors have read and approved the final version of the manuscript.

## CONFLICT OF INTEREST STATEMENT

The authors declare no conflict of interest.

## ETHICS STATEMENT

Ethical approval was granted by the local Ethics Committee of Technical University of Munich (IRB approval number 714/20 S). Informed consent was obtained from all patients prior to study inclusion.

## Data Availability

The datasets generated and/or analysed during the current study are available from the corresponding author on reasonable request.
